# Investigation of Roles of SLC38A1 in Proliferation and Differentiation of Mouse Tongue Epithelium and Expression in Human Oral Tongue Squamous Cell Carcinoma

**DOI:** 10.3390/cancers16020405

**Published:** 2024-01-18

**Authors:** Dipak Sapkota, Daxin Wang, Olaf Schreurs, Evan M. Vallenari, Sushma Pandey Dhakal, Thomas Küntziger, Burcu Sengüven Toközlü, Tor Paaske Utheim, Farrukh Abbas Chaudhry

**Affiliations:** 1 Department of Oral Biology, Faculty of Dentistry, University of Oslo, 0372 Oslo, Norway; 2 Department of Molecular Medicine, Institute of Basic Medical Sciences, University of Oslo, 0372 Oslo, Norway; 3 Department of Oral Pathology, Faculty of Dentistry, Gazi University, Ankara 06510, Turkey; 4 Department of Plastic and Reconstructive Surgery, Oslo University Hospital, 0372 Oslo, Norway

**Keywords:** head and neck cancer, oral cancer, maturation, glutamine, methylation, SNAT1, Slc38a1

## Abstract

**Simple Summary:**

Alteration of metabolic pathways, including the activation of aerobic glycolysis and increased dependence on glutamine, is characteristic of several human cancer types. Slc38a1 (also known as SNAT1, SAT1, SA2 or ATA1) is a cell membrane transporter highly capable of the net accumulation of cellular glutamine. It has been shown to be upregulated in several human cancers and/or associated with tumor progression. However, the functional role of this protein in the proliferation and differentiation of the oral epithelium, and its expression in human oral tongue squamous cell carcinoma (OTSCC), is not well understood. This study shows the upregulated expression of SLC38A1 in OTSCC indicating a role of SLC38A1 in OTSCC progression.

**Abstract:**

The aerobic glycolytic pathway, boosting lactate formation, and glutamine addiction are two hallmarks of cancer pathophysiology. Consistent with this, several cell membrane glutamine transporters, belonging to different solute carrier (SLC) families, have been shown to be upregulated in a cell-specific manner to furnish the cells with glutamine and glutamine-derived metabolic intermediates. Among them, the system A transporter Slc38a1 has a higher affinity for glutamine compared to other SLC transporters, and it undergoes highly multifaceted regulation at gene and protein levels. The current study aimed to investigate the functional role of Slc38a1 in the proliferation and maturation of the mouse tongue epithelium. Secondly, we aimed to examine the expression of *SLC38A1* and its regulation in human tongue oral squamous cell carcinoma (OTSCC). Employing *Slc38a1* wild-type and knockout mice, we showed that Slc38a1 was not directly linked to the regulation of the proliferation and differentiation of the mouse tongue epithelium. External transcriptomic datasets and Western blot analyses showed upregulation of *SLC38A1* mRNA/protein in human OTSCC and oral cancer cell lines as compared to the corresponding controls. Further, an investigation of external datasets indicated that mechanisms other than the amplification of the *SLC38A1* chromosomal locus or hypomethylation of the *SLC38A1* promoter region might be important for the upregulation of *SLC38A1* in OTSCC.

## 1. Introduction

Glutamine, the most abundant amino acid in the blood, functions as a precursor molecule for the synthesis of non-essential amino acids, proteins, nucleotides, the antioxidant glutathione and more, as well as provides key intermediates for the tricarboxylic acid cycle (TCA; also known as the Krebs cycle) [1]. Consistent with this, an increased requirement of glutamine has been demonstrated in rapidly dividing normal stem cells. Hence, the availability of an appropriate amount of glutamine and specialized membrane transporters to translocate glutamine across the membrane barrier and into cells and organelles is critical for normal cell structure and function [2,3].

It is now increasingly recognized that cancer cells display altered cellular metabolic pathways to support the growing tumor mass [4]. Among others, cancer cells have been shown to activate the aerobic glycolytic pathway, resulting in the augmented formation of lactate from pyruvate (Warburg effect) [5] and increased dependence on glutamine [6,7] to support their augmented energy and metabolite demands. In line with this, the upregulation of several glutamine transporters [8,9] and/or activation of upstream signaling pathways such as c-Myc [10] and K-Ras [11] have been demonstrated in several human cancers. Several transport systems pushing glutamine and other neutral amino acids across membranes have been demonstrated and classified as systems A, ASC, B, L and N [12]. These are now molecularly identified and organized into four Slc families, i.e., Slc1, Slc6, Slc7 and Slc38 [13,14].

The SLC38 family consists of 11 members that are involved in the transport of glutamine and/or other neutral amino acids across cell and organelle membranes [15]. We have demonstrated that several of the Slc38 isoforms have properties consistent with the classical system A and system N transporters and that they work in concert to shuttle glutamine intercellularly [1,16,17,18]. Interestingly, while most glutamine/amino acid transporters in all four Slc families make shallow gradients across cell membranes, including system ASC, L and N, the system A transporter Slc38a1 is able to provide cells with a net uptake of glutamine [19,20]. Slc38a1 is particularly suitable in glutamine accumulation when there is a high requirement because it works unidirectionally and it has the highest affinity for glutamine: the Km for glutamine is 0.37 mM at −50 mV in *Xenopus laevis* oocytes, which is much lower than that for any other transporter under similar conditions [19]. In addition, it has both narrow substrate specificity and cell-specific localization, such as in brain parvalbumin-expressing interneurons for the formation of the fast neurotransmitter GABA [18,19]. Furthermore, Slc38a1 is important for brain development and may define protein anabolism by regulating the activity of mTOR [20,21]. Consistent with these abilities, increased expression of SLC38A1 has been reported in several human malignancies such as gastric cancer [9], hepatocellular carcinoma [22] and malignant melanoma [23] as compared to the respective normal control tissues, indicating a link between SLC38A1 and human malignancies. Thus, SLC38A1 may be upregulated in cancer cells requiring consistently high levels of glutamine.

The functional role of Slc38a1 in the maturation process of the mouse tongue epithelium and its expression in human oral tongue squamous cell carcinoma (OTSCC), the most frequent type of oral squamous cell carcinoma (OSCC), remains to be explored. Here, we first showed that the proliferation and differentiation of the tongue epithelium are similar in *Slc38a1*^+/+^ and *Slc38a1*^−/−^ mice. The inactivation of *Slc38a1* in the mice tongue was not associated with significant compensatory upregulation of other *Slc38* gene members either. Rather, we detected the down-regulation of *Slc38a3* (also known as SN1 or SNAT3). An investigation of external transcriptomic datasets showed significant upregulation of *SLC38A1* mRNA in human OSCC as compared to the control oral mucosa, an observation most likely associated with mechanisms other than the amplification of the *SLC38A1* chromosomal locus or hypomethylation of the *SLC38A1* promoter region.

## 2. Materials and Methods

The outline of materials and methods is summarized in Supplementary Figure S1.

### 2.1. Cells

Primary normal oral keratinocytes (NOK1 and NOK2) were isolated from mucosal tissue obtained after third molar extractions [24] and cultured in keratinocyte serum-free medium as described previously [24,25]. The OSCC-derived cell lines CaLH3 [26] and SCC25 [27] were grown in a DMEM/F12 1:1 mixture (Gibco) supplemented with 10% FBS, 10 ng/mL epidermal growth factor, 0.4 µg/mL hydrocortisone, 0.05× ITS-A (Gibco), 50 µg/mL sodium L-ascorbate, 2% L-Glutamine and 1× antibiotic and antimycotic solution. All cells were grown in a humidified atmosphere of 5% CO_2_ in air at 37 °C. Isolation and the use of NOK were approved by the Regional Committees for Medical Research Ethics South East Norway (REK-2013/1818).

### 2.2. Animal Handling

Animal experiments were performed in accordance with EU and local regulations at the Department of Comparative Medicine, Institute of Basic Medical Sciences, Faculty of Medicine, University of Oslo. The use of animals in the current study was approved by the Norwegian Food Safety Authority (FOTS 21009). Wild-type (WT) C57BL/6 (*Slc38a1*^+/+^) and C57BL/6 knockout mice for Slc38a1 (*Slc38a1*^−/−^) were housed in GM500 or GM900 cages (Scanbur, Karlslunde, Denmark) in a temperature-controlled (22–26 °C) facility with 55 ± 10% humidity and a 12 h light/dark cycle. Mice were fed standard laboratory mouse chow (Ssniff GmbH, Soest, Germany) ad libitum and provided with plastic houses and paper enrichment. Mice genetically inactivated for Slc38a1 (*Slc38a1*^−/−^) were generated by excising exons 5–8 using the Cre-LoxP system in the C57BL/6 background [18]. The brains of these mice have been thoroughly characterized anatomically, biochemically and functionally in previous publications [18,28]. Three-month-old adult mice (*Slc38a1*^+/+^: *n* = 10 and *Slc38a1*^−/−^: *n* = 9), consisting of both males and females, were terminated via cervical dislocation. The anterior 2/3 of the tongue was dissected out, and a 2 mm segment of the apex of the tongue was removed and stored in RNA-free tubes at −80 °C. The remaining tissue was fixed in 10% neutral-buffered formalin for immunohistochemistry (IHC).

### 2.3. Measurement of Thickness of Tongue Epithelium in Mice

Blinded to the genotype status, the measurement of the epithelial thickness was performed by BST and DS. Hematoxylin and eosin-stained slides were scanned at 40× using a Pannoramic MIDI scanner (3DHISTECH, Budapest, Hungary), and digital images were analyzed in QuPath [29]. On a 400× magnification, using a 100 µm grid, a 400 µm long horizontal line was drawn across the region of interest (ROI) in the epithelium, and a polygon was drawn following the epithelial cells on the basal layer and the most superficial layer (excluding surface keratin) around the drawn line (Figure 2A). The average thickness of the epithelium was calculated by dividing the surface area of the drawn polygon by the length (400 µm). At least 2 ROI were measured both on the dorsal and ventral surfaces of the tongue.

### 2.4. RNA Isolation and Quantitative Real-Time Polymerase Chain Reaction (qRT-PCR)

Tissue stored in RNA later was thawed and transferred to a 2 mL round-bottomed tube with 1.4 mm ceramic beads and 350 µL buffer RLT (Cat. no. 79216, Qiagen, Hilden, Germany). The tissue was homogenized with 3 rounds of 5000 rpm for 45 s on a Precellys 24 tissue homogenizer. The samples were centrifuged at 12,000 rpm for 3 min at room temperature, and the supernatant was used for the isolation of RNA using the RNeasy Mini kit (Cat. no. 74104, Qiagen, Germany). The TURBO DNA-free™ Kit was used for removing contaminating DNA before converting RNA to cDNA. After genomic DNA removal, 1 µg of RNA per sample was used to synthesize cDNA using the High-Capacity RNA-to-cDNA™ Kit.

The relative quantitation of gene expression was performed by using real-time quantitative PCR (qRT-PCR) according to the ‘Guide to Performing Relative Quantitation of Gene Expression Using Real-Time Quantitative PCR’ from Applied Biosystems, and the PowerUp™ SYBR™ Green Master Mix kit (Applied Biosystems, Warrington, UK) was applied in this study. Briefly, relative standard curves for the housekeeping gene (Actb) as well as for several Slc38 genes of interest were created by using a series of diluted samples from mice, and the qRT-PCR primer efficiency was determined from the standard curve. The Pfaffl formula was used to calculate the gene expression ratio (GER), and the GERs of target genes were normalized to that of the housekeeping gene. The normalized values of *Slc38*a1^−/−^ were then compared to the mean values of WT samples. The statistics were performed in GraphPad Prism for Windows (https://www.graphpad.com/; version 9.4.1), and an unpaired *t*-test was used in this study.

### 2.5. Immunohistochemistry (IHC)

Four-microns-thick formalin-fixed paraffin-embedded (FFPE) sections were deparaffinized in xylene and hydrated in graded ethanol. Endogenous peroxidase was quenched in 0.3% hydrogen peroxide in methanol for 30 min before slides were washed in tap water. Heat-induced epitope retrieval was performed in Target Retrieval Solution Citrate pH 6 (Dako S2369, Glostrup, Denmark) for mice tissue, whereas an aqueous solution of 0.05% citraconic anhydride pH 7.4 (Sigma-Aldrich, St Louis, MO, USA) was used for human tissues. For the primary antibodies made in rabbit, the sections were blocked with 5% goat serum prior to incubation with the polyclonal anti-Slc38a1 antibody. Anti-Slc38a1 antibodies were generated against a glutathione S-transferase (GST)-fusion protein containing the N-terminal of Slc38a1 in rabbits. Following affinity purification, their specificity has been demonstrated in previous publications [18,28,30]. Besides the anti-Slc38a1 antibody, monoclonal anti-CK10 (clone EP1607IHCY, Abcam, Cambridge, UK) or anti-Ki-67 (clone SP6, Thermo Fisher Scientific, Waltham, MA, USA) antibodies were also used for IHC. After washing, the bound polyclonal antibody was amplified via incubation with undiluted EnVision+ horseradish peroxidase labeled polymer anti-rabbit (Dako), whereas the bound monoclonal antibodies were amplified with biotinylated goat-anti-rabbit IgG and horseradish peroxidase conjugated ABC reagent successively (Vector Labs).

For using mouse antibody on mouse tissue, sections were blocked for 1 h with 5% horse serum containing 13 µg/mL Fab fragment of donkey anti-mouse IgG (Jackson ImmunoResearch Labs, RRID:AB_2307338) prior to incubation with mouse monoclonal anti-CK4 (clone 6B10, Santa Cruz Biotechnology, Dallas, TX, USA). Bound antibodies were amplified with biotinylated horse-anti-mouse IgG for 10 min followed by horseradish peroxidase conjugated ABC reagent (Vector Laboratories, Newark, CA, USA). Primary and secondary antibodies were diluted in the Mouse on Mouse diluent (Vector Laboratories). The enzyme label was visualized by using 3,3′-diaminobenzidine and intensified with 0.5% copper(II) sulfate solution in saline. Nuclei were counterstained with Mayer’s hematoxylin (Dako) before the sections were dehydrated and embedded in Histokit (Karl Hecht, Sondheim vor der Rhön, Germany).

Incubations with primary antibodies were performed overnight at 4 °C. All other incubations were performed for 30 min at 22 °C unless otherwise specified. The slides were rinsed in phosphate-buffered saline (PBS) between the incubations, whereas dilutions were made in 1% IgG-free bovine serum albumin (Jackson ImmunoResearch Europe, Cambridgeshire, UK) in PBS. Chemicals, other than those stated above, were obtained from Sigma (Merck, Darmstadt, Germany).

Rat brain sections served as positive controls, while PBS instead of the primary antibody served as a negative control for the secondary antibody. Moreover, rabbit immunoglobulin fraction, X0903 (Dako, Glostrup, Denmark), was used to examine the nonspecific binding of the primary antibodies (isotype-matched control). Details of the antibodies used for IHC are presented in Table S1.

### 2.6. IHC Assessments of Mice Tongue Specimens

Blinded to the genotype status, IHC evaluation was performed by BT and DS. The scoring of Ki67 was carried out using a light microscope on a 40× magnification. All cells located on the basal layer of the tongue epithelium were counted. For each specimen, two different areas on each dorsal, ventral and lateral surface of the tongue were quantified. The proliferation index for each specimen was calculated as the percentage of positively stained cells per total number of epithelial cells on the basal layer. CK4 and CK10 were semiquantitatively scored into four groups: negative (−, no positive cells), weak (+, <10% positive cells), moderate (++, 10–70% positive cells) and strong (+++, >70% positive cells). 

### 2.7. External Transcriptomic and Genomic Data

Transcriptomic data for *SLC38A1* from head and neck squamous cell carcinoma (HNSCC; *n* = 519) and controls (*n* = 44) from The Cancer Genome Atlas (TCGA)/Genotype Tissue Expression (GTEx) [31] datasets were plotted using the Gene Expression Profiling Interactive Analysis (GEPIA) tool [32]. In addition, SLC38A1 mRNA levels for OSCC (*n* = 40) and pair-wise matched control mucosa were obtained from the GEO database (GSE37991) [33].

Amplification data for *SLC38A1* were exported from TCGA (Firhose legacy) using the cBioPortal tool [34]. Out of 504 cases of HNSCC, data for 132 cases of OTSCC were examined for the possible amplification of *SLC38A1*.

### 2.8. DNA Methylation Profiling

Promoter methylation data were accessed from NCBI GEO GSE75537 [35] (54 oral tongue primary tumors, 29 pair-wise matched normal control and 25 pair-wise matched peripheral blood). Following bisulfite conversion, DNA methylation at cytosine residues (5-methylcytosine; 5mC) was measured using an Infinium Human Methylation 450K BeadChip high-density array (Illumina). Data were analyzed with Geo2R (https://www.ncbi.nlm.nih.gov/geo/geo2r, accessed on 15 December 2022). Specific methylation profile graphs were generated using CpG island probes located in the *SLC38A1* promoter (cg16469386 and cg17726022) and in the housekeeping gene *GAPDH* promoter (cg00241355 and cg20917484) as a control. Methylation data were normalized based on the signal intensities beta distribution (total methylated/(total methylated + total unmethylated)).

### 2.9. Western Blotting

The tissues were homogenized in RIPA buffer, mixed with 0.1% SDS, applied to 10% Criterion™ TGX™ Precast Midi Protein Gel (BioRad, Hercules, CA, USA) for separation (90 V for 20 min and 220 V for 30 min) and electroblotted onto PVDF membranes (2.5 A, 25 V, 10 min) by using Trans-BlotTurbo Transfer System RTA Transfer Kits (BioRad, Hercules, CA, USA). The blots were incubated with 5% skimmed milk in TBST (0.05 M Tris-HCl, pH 7.4, 0.9% sodium chloride, and 0.1% Triton) to block unspecific staining followed by incubation with different primary antibodies in 3% skimmed milk in TBST overnight at 4 °C. After rinsing with TBST 3 × 10 min, the blots were incubated in secondary antibody conjugated with horseradish peroxidase for 1 h at room temperature. After rinsing with TBST 3 × 10 min, the signals of the blots were captured by using ChemiDoc Imaging Systems (BioRad, Hercules, CA, USA). To compensate for loading errors, the values were normalized to GAPDH staining of the same blots. For quantitation of the relevant bands, squares were made of the exact same size for each lane in Image Lab 6.0 (BioRad, Hercules, CA, USA), and the signal intensity was measured. The background was determined and eliminated by using the same square size outside the relevant protein band.

### 2.10. Statistics

Data are presented as mean ± standard deviation (SD) or standard error of the mean (SEM). The comparison of means between two unrelated and pair-wised matched groups was performed by using unpaired and paired Student’s *t*-tests, respectively. GraphPad Prism for Windows (https://www.graphpad.com/; version 9.4.1) was used for statistical analysis. A *p*-value of <0.05 was considered statistically significant.

## 3. Results

### 3.1. Slc38a1 mRNA and Protein Are Expressed in Normal and Malignant Oral Cells

**Figure 1:**
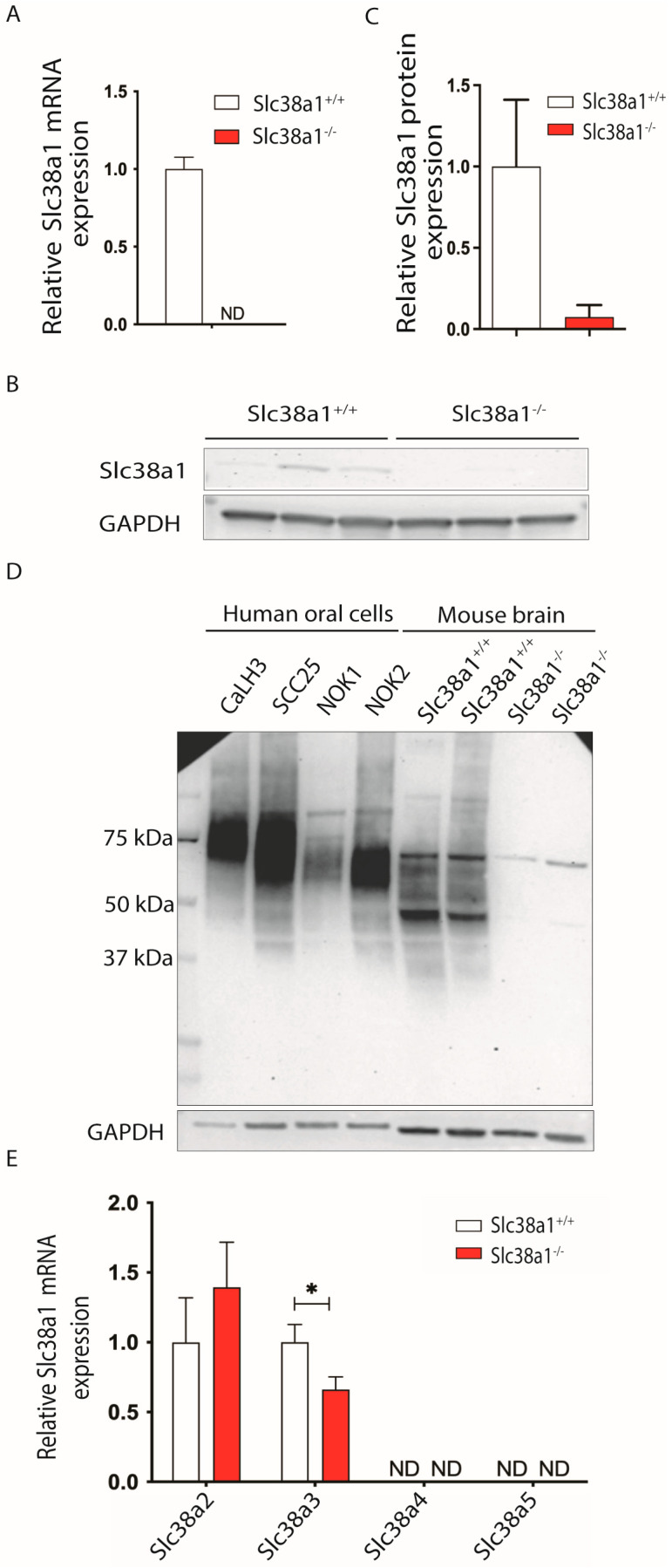
Mouse tongue specimens, normal oral keratinocytes and oral squamous cell carcinoma-derived cell lines express Slc38a1/SLC38A1 protein. (**A**) mRNA expression levels of Slc38a1 were examined in tongue specimens of *Slc38a1*^+/+^ (*n* = 10) and *Slc38a1*^−/−^ (*n* = 9) mice via real-time polymerase chain reaction (RT-PCR). *Slc38a1*^+/+^ mice revealed abundant *Slc38a1* mRNA, while no *Slc38a1* mRNA was detected in *Slc38a1*^−/−^ mice. Error bars represent SEM (unpaired *t*-test). (**B**) Slc38a1 protein levels were measured in tongue extracts (*n* = 3 for each genotype) separated via SDS-PAGE and immunolabeled with affinity-purified antibodies generated selectively towards Slc38a1. Western blot unveiled weak bands at ~50–55 kDa in tongue specimens of *Slc38a1*^+/+^ mice, whereas the corresponding expression in *Slc38a1*^−/−^ mice was imperceptible. Full blots are presented in Supplementary Figure S2. (**C**) Densitometric quantification of the Western blot data confirmed expression of Slc38a1 in *Slc38a1*^+/+^ mice, while expression in *Slc38a1*^−/−^ mice was negligible. Error bars represent SEM (unpaired *t*-test). (**D**) We investigated the expression of SLC38A1 in primary normal oral keratinocytes (NOK1 and NOK2) and oral squamous cell carcinoma-derived cell lines (CaLH3 and SCC25). Western blot demonstrated strongly labeled broad bands at ~50–80 kDa for SLC38A1 in all cell lysates. As a control, brain extracts from *Slc38a1*^+/+^ and *Slc38a1*^−/−^ mice were included in the same experiments. *Slc38a1*^+/+^ brain extracts showed broad stained bands at ~45–65 kDa. In *Slc38a1*^−/−^ mice, only a weak slender band was seen at ~65–70 kDa. (**E**) We next investigated the expression of closely related Slc38-isoforms in *Slc38a1*^−/−^ and wild-type mice. *Slc38a2* and *Slc38a3* mRNA were detected in normal tongue tissue, while no transcripts of *Slc38a4* and *Slc38a5* could be detected. Upon genetic inactivation of *Slc38a1*, *Slc38a3* was found to be significantly down-regulated compared to the wild-type mice, while *Slc38a2* was moderately but not significantly upregulated. Error bars represent SEM (unpaired *t*-test). * represents <0.05 p-value. ND, not detected.

In order to investigate the possible function of Slc38a1 in the oral epithelium, we investigated the expression of the *Slc38a1* transcript in normal mouse tongues. qRT-PCR showed that *Slc38a1*^+/+^ mice expressed *Slc38a1* mRNA in tongue specimens, while *Slc38a1*^−/−^ is devoid of the *Slc38a1* transcript (Figure 1A). Expression of *Slc38a1* mRNA in tongue specimens from *Slc38a1*^+/+^ mice suggests a potential role of Slc38a1 in the oral epithelium. It also supports the specificity of the qRT-PCR. We next tested whether the *Slc38a1* was translated in situ, as many transcripts do not make it into functional proteins. Western blotting revealed a single weak band at ~50–55 kDa in *Slc38a1*^+/+^ (Figure 1B,C and Figure S2), consistent with our previous studies demonstrating Slc38a1-specific bands in mouse and rat brains [18,30]. The specificity of Western blotting is further supported by barely any bands on immunoblots from *Slc38a1*^−/−^ mouse tongue tissue (Figure 1B,C). Thus, both the *Slc38a1* transcript and protein were expressed selectively in *Slc38a1*^+/+^ mice, suggesting a functional role for Slc38a1 in the mouse tongue epithelium.

Next, we investigated whether SLC38A1 was expressed in normal human oral epithelial (NOK1 and NOK2) and malignant (CaLH3 and SCC25) cells. Western blot using affinity-purified antibodies against Slc38a1 demonstrated strongly labeled broad bands at ~50–80 kDa for SLC38A1 in cell lysates from all of the cell lines examined (Figure 1D). The higher molecular weight of the bands in these cells is consistent with cell-specific post-translational modifications, as we have shown for several isoforms of the Slc38 family in rat brain, liver, kidney and pancreas [36,37]. Of note, the bands were stronger in malignant oral cells as compared to the normal oral keratinocytes (Figure 1D). The specificity of the Slc38a1 antibody was corroborated by including immunoblotting for Slc38a1 in two brain samples from *Slc38a1*^+/+^ and two from *Slc38a1*^−/−^ mice (Figure 1D). Consistent with our previous results [18,30], Western blot analysis showed a broad stained band at ~45–65 kDa in brain tissues in *Slc38a1*^+/+^ mice (Figure 1D). This broad band is not observed in the brain tissue of *Slc38a1*^−/−^ mice. The thin sharp band at ~65–70 kDa in the homogenates of *Slc38a1*^−/−^ is also seen in *Slc38a1*^+/+^ mice and likely represents a spurious band (Figure 1D). Altogether, our data demonstrate the expression of SLC38A1 in normal and malignant oral cells.

### 3.2. Inactivation of Slc38a1 Gene Was Associated with Significant Down-Regulation of Slc38a3 in Mouse Tongue

We tested whether four other main system A and system N transporters involved in intercellular glutamine shuttling were also expressed in mouse tongue tissues. *Slc38a2* and *Slc38a3* mRNAs were found to be expressed in tongue tissues, while *Slc38a4* and *Slc38a5* expression was not detected in *Slc38a1*^+/+^ mice (Figure 1E). Next, we wanted to examine if the knockout of *Slc38a1* was associated with any alteration in the expression of other *Slc38* gene members to compensate for the loss of Slc38a1. Genetic inactivation of *Slc38a1* was associated with significant down-regulation of *Slc38a3* and moderate but not significant upregulation of *Slc38a2* in *Slc38a1*^−/−^ mouse tongue tissue as compared to *Slc38a1*^+/+^ mouse tissue (Figure 1E). A disruption of *Slc38a1* did not impact *Slc38a4* and *Slc38a5* mRNA levels in mouse tongue tissues (Figure 1E).

### 3.3. Slc38a1^+/+^ and Slc38a1^−/−^ Mice Showed No Significant Difference in Epithelial Thickness and Proliferation and Differentiation Pattern of Tongue Epithelium

**Figure 2:**
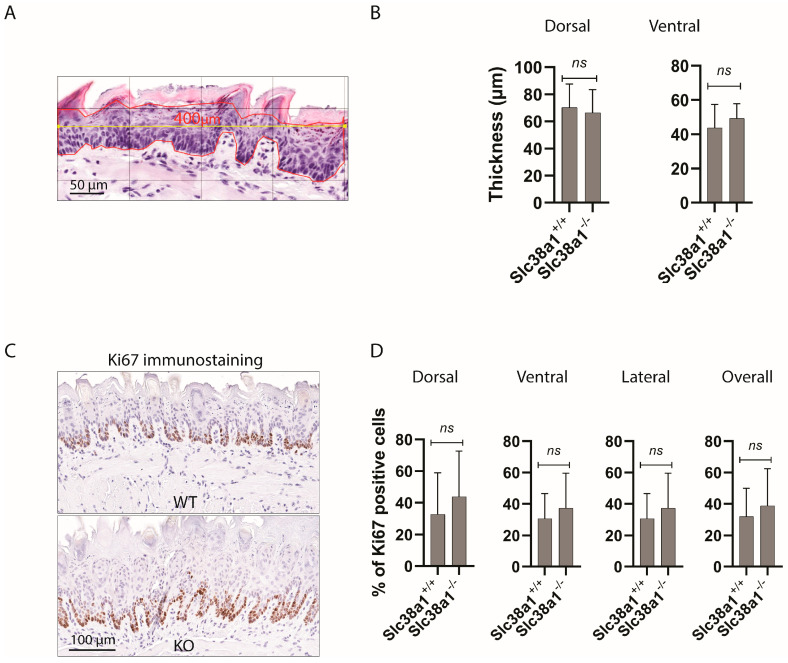
Genetic inactivation of *Slc38a1* was not associated with significant changes in epithelial thickness or the proliferation and/or differentiation pattern of the tongue epithelium in mice. (**A**) The thickness of the epithelium was measured by selecting a region of interest (ROI) under 400× magnification and using a 100 µm grid. A 400 µm long horizontal line (marked in yellow) was drawn across the ROI, and a polygon (marked in red) was drawn following the epithelial cells on the basal layer and the most superficial layer (excluding the surface keratin). The average thickness of the epithelium was calculated by dividing the surface area of the drawn polygon by the length (400 µm). (**B**) The epithelial thickness both on the dorsal and ventral surfaces of mouse tongues was similar in *Slc38a1*^+/+^ and *Slc38a1*^−/−^ mice. (**C**) The proliferation index was measured by Ki67 immunostaining in *Slc38a1*^−/−^ and *Slc38a1*^+/+^ mice. The figure shows representative images of Ki67 immunostaining on the dorsal surface of tongues from *Slc38a1*^+/+^ (WT) and *Slc38a1*^−/−^ (KO) mice. (**D**) Quantification of Ki67 immunolabeling in the dorsal, ventral and lateral surfaces of tongues from *Slc38a1*^+/+^ (*n* = 10) and *Slc38a1*^−/−^ (*n* = 9) mice was performed. No significant changes were seen in any of the surfaces upon comparison of the two genotypes. Unpaired Student’s *t*-test was used for the statistical analysis. Error bars represent SD.

We tested whether the expression of Slc38a1 was related to the thickness of the mouse tongue epithelium. Morphometric analysis (as described in Figure 2A) showed that there was no significant difference between *Slc38a1*^+/+^ and *Slc38a1*^−/−^ mice in the epithelial thickness of neither the dorsal nor the ventral surfaces of the tongue (Figure 2A,B). We next scrutinized whether Slc38a1 affects the cellular proliferation and/or differentiation of the tongue epithelium in mice using immunostaining for Ki67, CK4 and CK10, the markers for proliferation and differentiation. No significant difference in the immunoexpression of Ki67, CK4 and CK10 in the tongue epithelium was observed between *Slc38a1*^+/+^ and *Slc38a1*^−/−^ mice (Figure 2C,D and Table 1).

### 3.4. SLC38A1 mRNA Levels Are Upregulated in OSCC/HNSCC

**Figure 3:**
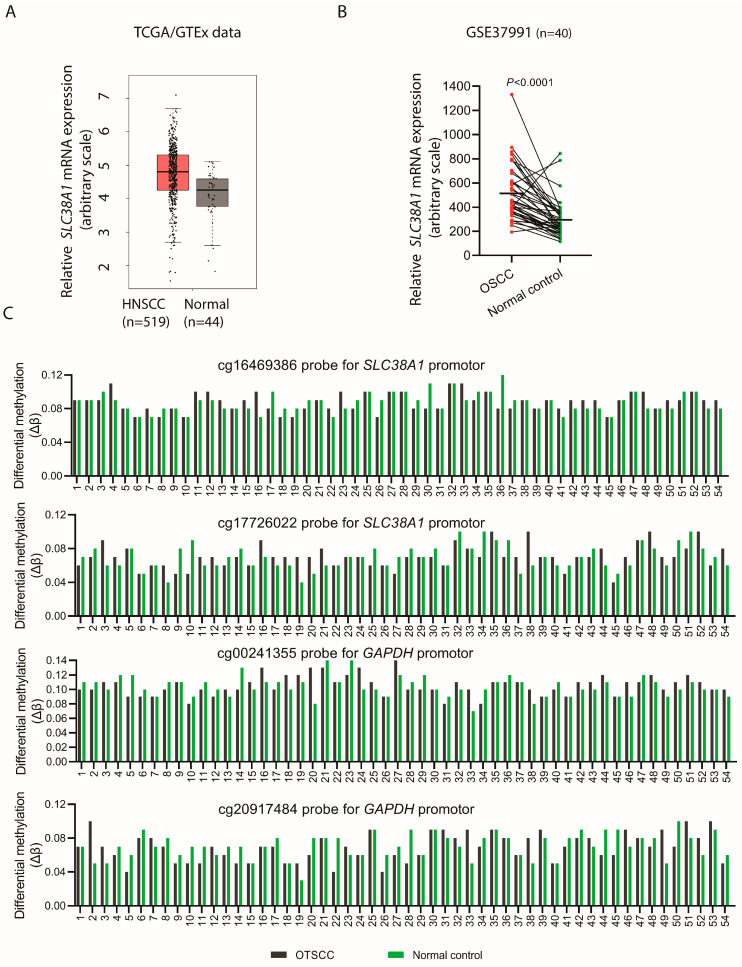
*SLC38A1* mRNA was upregulated in head and neck squamous cell carcinoma (HNSCC) and in oral squamous cell carcinoma (OSCC) specimens, but promoter methylation status was not changed in oral tongue squamous cell carcinomas. Analysis of transcriptome datasets showed upregulation of *SLC38A1* mRNA levels in HNSCC (*p* > 0.05) (**A**) and OSCC (*p* < 0.0001) (**B**) specimens as compared to the corresponding controls. (**C**) Analysis of promoter DNA methylation data from 54 OTSCC with pair-wise matched controls using probes located in the *SLC38A1* (cg16469386 and cg17726022) and *GAPDH* (cg00241355 and cg20917484) promoter regions. For each probe, methylation is quantified in all samples by expressing the methylated/(methylated + unmethylated) signal intensity ratio.

As the malignant oral epithelial cells CaLH3 and SCC25 showed robust expression of SLC38A1 as compared to the normal oral keratinocytes, we hypothesized that *SLC38A1* was upregulated in carcinomas as compared to the corresponding control specimens. For this, we examined the mRNA expression of *SLC38A1* in OSCC and HNSCC in two different transcriptomic datasets. *SLC38A1* mRNA was indeed upregulated in the carcinomas compared to normal controls (Figure 3A,B).

### 3.5. SLC38A1 Gene Was Not Amplified in OTSCC Specimens

The significant upregulation of *SLC38A1* mRNA levels in oral carcinomas as compared to normal control specimens prompted us to investigate the possible amplification of the *SLC38A1* gene in OTSCC species. An investigation of 132 cases of OTSCC from TCGA revealed no *SLC38A1* gene amplification.

### 3.6. Methylation Status of SLC38A1 Gene Promoter in OTSCC Was Similar as Compared to Normal Control Oral Epithelium

The examination of the methylation status of the *SLC38A1* gene promoter using public datasets showed a similar methylation status profile (*p* > 0.05) both in OTSCCs (*n* = 54) and in corresponding pair-wise matched normal control specimens using two different CpG island probes (cg16469386 and cg17726022) (Figure 3C). A similar methylation status was observed in the promoter regions (cg00241355 and cg20917484 probes) of *GAPDH* both in the OTSCC and corresponding normal control specimens.

## 4. Discussion

Given the key role of glutamine in the homeostasis of epithelial cells [38], including the oral epithelium [39], the current study investigated the role of Slc38a1, one of the key glutamine transporters, in the proliferation and differentiation of the oral tongue epithelium in mice. The tongue epithelium thickness (both at the dorsal and ventral surfaces) of *Slc38a1*^−/−^ mice was found to be similar to that of *Slc38a1*^+/+^ mice. One of the main determinants of epithelial thickness is the rate of keratinocyte proliferation. In line with the above observation, further experiments demonstrated similar proliferation indices (Ki67 labeling) of tongue keratinocytes both in *Slc38a1*^−/−^ and *Slc38a1*^+/+^ mice. Besides proliferation, cellular differentiation also contributes to the epithelial structure and thickness. The immunoexpression of CK4 and CK10 was found to be similar both on the dorsal and ventral surfaces of the tongue in *Slc38a1*^−/−^ and *Slc38a1*^+/+^ mice. Altogether, these results do not support a direct functional role of Slc38a1 in regulating the proliferation and differentiation of the epithelium in mouse tongue, as has been previously reported for the neuronal proliferation and differentiation of pluripotent P19 cells [40]. Nevertheless, this difference could be related to cell-tissue-specific functions of Slc38a1. In addition, epithelial and other cells have many other glutamine transporters and proteins that may be upregulated and/or compensate for the loss of Slc38a1 as we previously reported in the mouse brain [18].

Both SLC38A1 and SLC38A2 represent the classical transport system A activity, which is highly regulated, e.g., manifested during cell growth and amino acid deficiency [41]. Previous studies have reported the upregulation of SLC38A2 in cancer cells with knock-down of the *SLC38A1* gene, indicating a possible compensatory functional overlap between SLC38 protein members [42]. In harmony with these data, we found moderate, but not significant, upregulation of *Slc38a2* in *Slc38a1*^−/−^ mouse tongue tissue as compared to that of *Slc38a1*^+/+^ mouse. Indeed, SLC38A2 is more suitable for glutamine uptake under stress conditions [43]. In contrast, we found significant down-regulation of *Slc38a3* mRNA levels in *Slc38a1*^−/−^ mouse tongue. We have previously shown that the system A transporters and system N transporters work in concert to shuttle glutamine between cells and organs [44]. In the brain, Slc38a1 and Slc38a3 shuttle glutamine from astroglial cells to GABAergic neurons for GABA synthesis, and, in the pancreas, Slc38a2 and Slc38a3 work in concert to regulate insulin secretion [28,37,44]. Thus, Slc38a3 may be down-regulated upon disrupted Slc38a1-Slc38a3 concerted function. This warrants further studies to clarify these suggestions.

Similar to other human malignancies such as gastric cancer [9], hepatocellular carcinoma [22] and malignant melanoma [23], *SLC38A1* mRNA expression was found to be upregulated in OSCC/HNSCC compared to the normal control tissues in the external transcriptomic datasets. In parallel, the expression of SLC38A1 was found to be higher in two OSCC cell lines (CaLH3 and SCC25) as compared to two normal oral keratinocytes. These results support a link between SLC38A1 and OSCC progression. This suggestion is in line with the observation by Sutinen and coauthors who demonstrated the uptake of [N-methyl-11C]α-methylaminoisobutyric acid (MeAIB), a prototypic substrate for system A transport, in HNSCC by using positron emission tomography scans [45].

Several mechanisms, such as epigenetic changes, including methylation status of the promoter region, gene amplification, miRNA-mediated gene regulation or transcription control via upstream signaling pathways, could be involved in the upregulation of *SLC38A1* in OSCC. An investigation of the externally available datasets showed no amplification of *SLC38A1* in OTSCC and a similar methylation status profile in the promoter region of *SLC38A1* both in OTSCCs and in corresponding pair-wise matched normal control specimens. Thus, other mechanisms might contribute to the upregulated expression in OSCC. Recently, a number of papers have demonstrated the miRNA-mediated regulation of SLC38A1 in human malignancies [46,47]. However, the possible role of miRNA in the regulation of SLC38A1 in OSCC needs further investigation.

Given the dependency of cancer cells on glutamine to support the energy and metabolite demand of the cancer cells and the concurrent activation of signaling pathways such as c-Myc [10] and K-Ras [11] in several cancer types, the possible activation of upstream signaling pathways/transcription factors might be a likely mechanism for the upregulation of SLC38A1 in OSCC. Indeed, the system A transporters Slc38a1 and Slc38a2 undergo multifaceted regulation, and their promoters are activated by c-Myc and ATF4 [20,48,49].

Several SLC38 isoforms have previously been linked to cell proliferation, invasion, migration and/or angiogenesis, including SLC38A2 [50], SLC38A3 [51], SLC38A6 [52] and SLC38A7 [53], supporting their involvement in cancer progression. Moreover, as Slc38a1 has a high affinity for glutamine compared to other glutamine transporters, and it transports glutamine unidirectionally [19,20], cells expressing SLC38A1 will be better suited for the accumulation of high levels of glutamine intracellularly and accompanying boosted cellular metabolism, which is favorable for cancer progression. In the same line, Böhme-Schäfer and coauthors have reported that SLC38A1 expression is associated with primary and metastatic cell lines and increased proliferation, colony formation, migration and invasion abilities of malignant melanoma cells [23].

## 5. Conclusions

In conclusion, although the inactivation of Slc38a1 does not seem to directly affect the proliferation and differentiation of the tongue epithelium, the upregulated expression of SLC38A1 in OSCC indicates a role of SLC38A1 in OSCC progression, perhaps reflecting the increased glutamine metabolism in OSCC cells. The findings of the current study form the basis for future functional and prognostic studies on SLC38A1 in OSCC.

## Figures and Tables

**Table 1:** Expression of CK4 and CK10 in mouse tongue epithelium.

		CK4		CK10	
Sample	Genotype	Dorsal	Ventral	Dorsal	Ventral
16A	WT	+	−	+++	+++
13A	WT	+	+	++	+++
12A	WT	+	−	+	++
14A	WT	+	+	+++	+++
26A	WT	+	−	++	+++
29A	WT	++	+	+++	+++
19A	WT	+	+	+++	+++
30A	WT	+	−	+++	+++
24A	WT	+	+	+++	+++
23A	WT	+	−	+++	+++
15A	KO	++	+	++	+++
18A	KO	++	+	+++	+++
17A	KO	++	+	+++	+++
11A	KO	+	−	++	+++
21A	KO	+	−	NA	NA
28A	KO	++	+	+++	+++
27A	KO	+	+	+++	+++
20A	KO	+	+	+++	+++
31A	KO	+	−	+++	+++

NA: not available; WT: wild–type; KO: knockout; −: negative; +: weak; ++: moderate; +++: strong.

## Data Availability

Publicly available datasets used in the current study can be found here: NCBI GEO GSE75537 [35], TCGA/GTEx [31], GSE37991 [33] and The Cancer Genome Atlas (TCGA, Firhose legacy) through the cBioPortal tool [34]. Other data in this study are available upon request from the corresponding author.

## Supplementary Materials

The following supporting information can be downloaded at: https://www.mdpi.com/article/10.3390/cancers16020405/s1. Table S1: Details of antibodies used for immunohistochemistry; Figure S1: Flowchart summarizing the materials and methods used in the study; Figure S2: Full blots for Slc38a1 and Gapdh using lysates from tongue extracts from *Slc38a1^+/+^* and *Slc38a1^−/−^* mice.

## References

[1] Chaudhry F.A., Reimer R.J., Edwards R.H. (2002). The glutamine commute: Take the N line and transfer to the A. J. Cell Biol..

[2] Yu Y., Newman H., Shen L., Sharma D., Hu G., Mirando A.J., Zhang H., Knudsen E., Zhang G.-F., Hilton M.J. (2019). Glutamine metabolism regulates proliferation and lineage allocation in skeletal stem cells. Cell Metab..

[3] Marsboom G., Zhang G.-F., Pohl-Avila N., Zhang Y., Yuan Y., Kang H., Hao B., Brunengraber H., Malik A.B., Rehman J. (2016). Glutamine Metabolism Regulates the Pluripotency Transcription Factor OCT4. Cell Rep..

[4] Hanahan D., Weinberg R.A. (2011). Hallmarks of cancer: The next generation. Cell.

[5] Warburg O., Wind F., Negelein E. (1927). The metabolism of tumors in the body. J. Gen. Physiol..

[6] DeBerardinis R.J., Cheng T. (2010). Q’s next: The diverse functions of glutamine in metabolism, cell biology and cancer. Oncogene.

[7] Wise D.R., Thompson C.B. (2010). Glutamine addiction: A new therapeutic target in cancer. Trends Biochem. Sci..

[8] Wang K., Cao F., Fang W., Hu Y., Chen Y., Ding H., Yu G. (2013). Activation of SNAT1/SLC38A1 in human breast cancer: Correlation with p-Akt overexpression. BMC Cancer.

[9] Xie J., Li P., Gao H.-F., Qian J.-X., Yuan L.-Y., Wang J.-J. (2014). Overexpression of SLC38A1 is associated with poorer prognosis in Chinese patients with gastric cancer. BMC Gastroenterol..

[10] Wise D.R., DeBerardinis R.J., Mancuso A., Sayed N., Zhang X.-Y., Pfeiffer H.K., Nissim I., Daikhin E., Yudkoff M., McMahon S.B. (2008). Myc regulates a transcriptional program that stimulates mitochondrial glutaminolysis and leads to glutamine addiction. Proc. Natl. Acad. Sci. USA.

[11] Gaglio D., Metallo C.M., Gameiro P.A., Hiller K., Danna L.S., Balestrieri C., Alberghina L., Stephanopoulos G., Chiaradonna F. (2011). Oncogenic K-Ras decouples glucose and glutamine metabolism to support cancer cell growth. Mol. Syst. Biol..

[12] Barker G., Ellory J. (1990). The identification of neutral amino acid transport systems. Exp. Physiol..

[13] Bhutia Y.D., Ganapathy V. (2016). Glutamine transporters in mammalian cells and their functions in physiology and cancer. Biochim. Biophys. Acta (BBA)—Mol. Cell Res..

[14] Pochini L., Scalise M., Galluccio M., Indiveri C. (2014). Membrane transporters for the special amino acid glutamine: Structure/function relationships and relevance to human health. Front. Chem..

[15] Schiöth H.B., Roshanbin S., Hägglund M.G.A., Fredriksson R. (2013). Evolutionary origin of amino acid transporter families SLC32, SLC36 and SLC38 and physiological, pathological and therapeutic aspects. Mol. Asp. Med..

[16] Chaudhry F.A., Reimer R.J., Krizaj D., Barber D., Storm-Mathisen J., Copenhagen D.R., Edwards R.H. (1999). Molecular analysis of system N suggests novel physiological roles in nitrogen metabolism and synaptic transmission. Cell.

[17] Jenstad M., Chaudhry F. (2013). The amino acid transporters of the glutamate/GABA-glutamine cycle and their impact on insulin and glucagon secretion. Front. Endocrinol..

[18] Qureshi T., Sørensen C., Berghuis P., Jensen V., Dobszay M.B., Farkas T., Dalen K.T., Guo C., Hassel B., Utheim T.P. (2019). The glutamine transporter Slc38a1 regulates GABAergic neurotransmission and synaptic plasticity. Cereb. Cortex.

[19] Chaudhry F.A., Schmitz D., Reimer R.J., Larsson P., Gray A.T., Nicoll R., Kavanaugh M., Edwards R.H. (2002). Glutamine uptake by neurons: Interaction of protons with system A transporters. J. Neurosci..

[20] Menchini R.J., Chaudhry F.A. (2019). Multifaceted regulation of the system A transporter Slc38a2 suggests nanoscale regulation of amino acid metabolism and cellular signaling. Neuropharmacology.

[21] Yamada D., Kawabe K., Tosa I., Tsukamoto S., Nakazato R., Kou M., Fujikawa K., Nakamura S., Ono M., Oohashi T. (2019). Inhibition of the glutamine transporter SNAT1 confers neuroprotection in mice by modulating the mTOR-autophagy system. Commun. Biol..

[22] Liu Y., Yang Y., Jiang L., Xu H., Wei J. (2021). High expression levels of SLC38A1 are correlated with poor prognosis and defective immune infiltration in hepatocellular carcinoma. J. Oncol..

[23] Böhme-Schäfer I., Lörentz S., Bosserhoff A.K. (2022). Role of amino acid transporter SNAT1/SLC38A1 in human melanoma. Cancers.

[24] Schreurs O., Karatsaidis A., Balta M.G., Grung B., Hals E.K.B., Schenck K. (2020). Expression of keratins 8, 18, and 19 in epithelia of atrophic oral lichen planus. Eur. J. Oral Sci..

[25] Sengüven Toközlü B., Sapkota D., Vallenari E.M., Schreurs O., Søland T.M. (2023). Cortactin expression in a Norwegian cohort of human papilloma virus negative oral squamous cell carcinomas of the mobile tongue. Eur. J. Oral Sci..

[26] Harper L.J., Piper K., Common J., Fortune F., Mackenzie I.C. (2007). Stem cell patterns in cell lines derived from head and neck squamous cell carcinoma. J. Oral Pathol. Med..

[27] Rheinwald J.G., Beckett M.A. (1981). Tumorigenic keratinocyte lines requiring anchorage and fibroblast support cultured from human squamous cell carcinomas. Cancer Res..

[28] Qureshi T., Bjørkmo M., Nordengen K., Gundersen V., Utheim T.P., Watne L.O., Storm-Mathisen J., Hassel B., Chaudhry F.A. (2020). Slc38a1 conveys astroglia-derived glutamine into GABAergic interneurons for neurotransmitter GABA synthesis. Cells.

[29] Bankhead P., Loughrey M.B., Fernández J.A., Dombrowski Y., McArt D.G., Dunne P.D., McQuaid S., Gray R.T., Murray L.J., Coleman H.G. (2017). QuPath: Open source software for digital pathology image analysis. Sci. Rep..

[30] Solbu T.T., Bjørkmo M., Berghuis P., Harkany T., Chaudhry F. (2010). SAT1, a glutamine transporter, is preferentially expressed in GABAergic neurons. Front. Neuroanat..

[31] GTEx (2015). The genotype-tissue expression (GTEx) pilot analysis: Multitissue gene regulation in humans. Science.

[32] Tang Z., Li C., Kang B., Gao G., Li C., Zhang Z. (2017). GEPIA: A web server for cancer and normal gene expression profiling and interactive analyses. Nucleic Acids Res..

[33] Lee C.-H., Wong T.-S., Chan J.Y.-W., Lu S.-C., Lin P., Cheng A.-J., Chen Y.-J., Chang J.S.-M., Hsiao S.-H., Leu Y.-W. (2013). Epigenetic regulation of the X-linked tumour suppressors BEX1 and LDOC1 in oral squamous cell carcinoma. J. Pathol..

[34] Cerami E., Gao J., Dogrusoz U., Gross B.E., Sumer S.O., Aksoy B.A., Jacobsen A., Byrne C.J., Heuer M.L., Larsson E. (2012). The cBio cancer genomics portal: An open platform for exploring multidimensional cancer genomics data. Cancer Discov..

[35] Krishnan N.M., Dhas K., Nair J., Palve V., Bagwan J., Siddappa G., Suresh A., Kekatpure V.D., Kuriakose M.A., Panda B. (2016). A minimal DNA methylation signature in oral tongue squamous cell carcinoma links altered methylation with tumor attributes. Mol. Cancer Res..

[36] Solbu T.T., Boulland J.-L., Zahid W., Lyamouri Bredahl M.K., Amiry-Moghaddam M., Storm-Mathisen J., Roberg B.A., Chaudhry F.A. (2005). Induction and targeting of the glutamine transporter SN1 to the basolateral membranes of cortical kidney tubule cells during chronic metabolic acidosis suggest a role in pH regulation. J. Am. Soc. Nephrol..

[37] Gammelsaeter R., Jenstad M., Bredahl M.K.L., Gundersen V., Chaudhry F.A. (2009). Complementary expression of SN1 and SAT2 in the islets of Langerhans suggests concerted action of glutamine transport in the regulation of insulin secretion. Biochem. Biophys. Res. Commun..

[38] Kim M.-H., Kim H. (2017). The roles of glutamine in the intestine and its implication in intestinal diseases. Int. J. Mol. Sci..

[39] Hu J., Ling Z., Li W., Su Z., Lu J., Zeng Q., Cheng B., Tao X. (2023). Glutamine promotes the proliferation of epithelial cells via mTOR/S6 pathway in oral lichen planus. J. Oral Pathol. Med..

[40] Ogura M., Kakuda T., Takarada T., Nakamichi N., Fukumori R., Kim Y.-H., Hinoi E., Yoneda Y. (2012). Promotion of both proliferation and neuronal differentiation in pluripotent P19 cells with stable overexpression of the glutamine transporter slc38a1. PLoS ONE.

[41] Palacin M., Estevez R., Bertran J., Zorzano A. (1998). Molecular biology of mammalian plasma membrane amino acid transporters. Physiol. Rev..

[42] Bröer A., Rahimi F., Bröer S. (2016). Deletion of amino acid transporter ASCT2 (SLC1A5) reveals an essential role for transporters SNAT1 (SLC38A1) and SNAT2 (SLC38A2) to sustain glutaminolysis in cancer cells. J. Biol. Chem..

[43] Morotti M., Bridges E., Valli A., Choudhry H., Sheldon H., Wigfield S., Gray N., Zois C.E., Grimm F., Jones D. (2019). Hypoxia-induced switch in SNAT2/SLC38A2 regulation generates endocrine resistance in breast cancer. Proc. Natl. Acad. Sci. USA.

[44] Nissen-Meyer L.S., Chaudhry F. (2013). Protein kinase C phosphorylates the system N glutamine transporter SN1 (Slc38a3) and regulates Its membrane trafficking and degradation. Front. Endocrinol..

[45] Sutinen E., Jyrkkiö S., Alanen K., Någren K., Minn H. (2003). Uptake of [N-methyl-11C]α-methylaminoisobutyric acid in untreated head and neck cancer studied by PET. Eur. J. Nucl. Med. Mol. Imaging.

[46] Su Q., Wang H. (2021). Long non-coding RNA 01559 mediates the malignant phenotypes of hepatocellular carcinoma cells through targeting miR-511. Clin. Res. Hepatol. Gastroenterol..

[47] Cai C., Lin J., Li J., Wang X.-D., Xu L.-M., Chen D.-Z., Chen Y.-P. (2022). miRNA-432 and SLC38A1 as predictors of hepatocellular carcinoma complicated with alcoholic steatohepatitis. Oxidative Med. Cell. Longev..

[48] Shroff E.H., Eberlin L.S., Dang V.M., Gouw A.M., Gabay M., Adam S.J., Bellovin D.I., Tran P.T., Philbrick W.M., Garcia-Ocana A. (2015). MYC oncogene overexpression drives renal cell carcinoma in a mouse model through glutamine metabolism. Proc. Natl. Acad. Sci. USA.

[49] Tameire F., Verginadis I.I., Leli N.M., Polte C., Conn C.S., Ojha R., Salas Salinas C., Chinga F., Monroy A.M., Fu W. (2019). ATF4 couples MYC-dependent translational activity to bioenergetic demands during tumour progression. Nat. Cell Biol..

[50] Morotti M., Zois C.E., El-Ansari R., Craze M.L., Rakha E.A., Fan S.-J., Valli A., Haider S., Goberdhan D.C.I., Green A.R. (2021). Increased expression of glutamine transporter SNAT2/SLC38A2 promotes glutamine dependence and oxidative stress resistance, and is associated with worse prognosis in triple-negative breast cancer. Br. J. Cancer.

[51] Wang Y., Fu L., Cui M., Wang Y., Xu Y., Li M., Mi J. (2017). Amino acid transporter SLC38A3 promotes metastasis of non-small cell lung cancer cells by activating PDK1. Cancer Lett..

[52] Huang L., Li L., Cheng B., Xing T. (2022). SLC38A6, regulated by EP300-mediated modifications of H3K27ac, promotes cell proliferation, glutamine metabolism and mitochondrial respiration in hepatocellular carcinoma. Carcinogenesis.

[53] Verdon Q., Boonen M., Ribes C., Jadot M., Gasnier B., Sagné C. (2017). SNAT7 is the primary lysosomal glutamine exporter required for extracellular protein-dependent growth of cancer cells. Proc. Natl. Acad. Sci. USA.

