# Supplementary material for: Investigation of Roles of SLC38A1 in Proliferation and Differentiation of Mouse Tongue Epithelium and Expression in Human Oral Tongue Squamous Cell Carcinoma
Source: Cancers (Basel). 2024 Jan 18;16(2):405. doi: 10.3390/cancers16020405 (PMC10814082; doi:10.3390/cancers16020405)

Supplementary Table S1: Details of antibodies used for immunohistochemistry

Primary antibody	Source	RRID*	Amplification system
mAb CK4 clone 6B10, Santa Cruz Biotechnology	Mouse	AB_2249751	ABC-HRP, Vector labs
CK10 clone EP1607IHCY, Abcam	Rabbit	AB_1523465	ABC-HRP, Vector labs
mAb Ki-67 clone SP6, NeoMarkers	Rabbit	AB_2341197	ABC-HRP, Vector labs

*RRID: Research Resource Identification

Supplementary Figure S1: Flowchart summarizing the materials and methods used in the study.

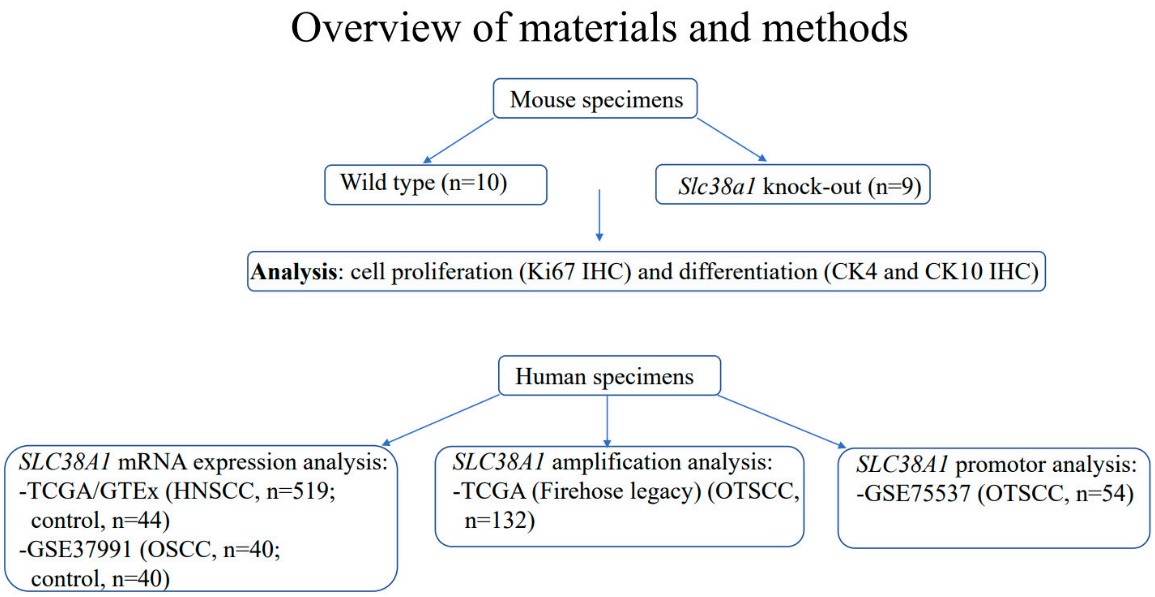

IHC: immunohistochemistry; TCGA/GTEx: The Cancer Genome Atlas/Genotype Tissue Expression; HNSCC: Head and Neck Squamous Cell Carcinoma; OSCC: Oral Squamous Cell Carcinoma; OTSCC: Oral Tongue Squamous Cell Carcinoma

Supplementary Figure S2: Full blots for Slc38a1 and Gapdh using lysates from tongue extracts from *Slc38a1*$^{+/+}$ and *Slc38a1*$^{-/-}$ mice.

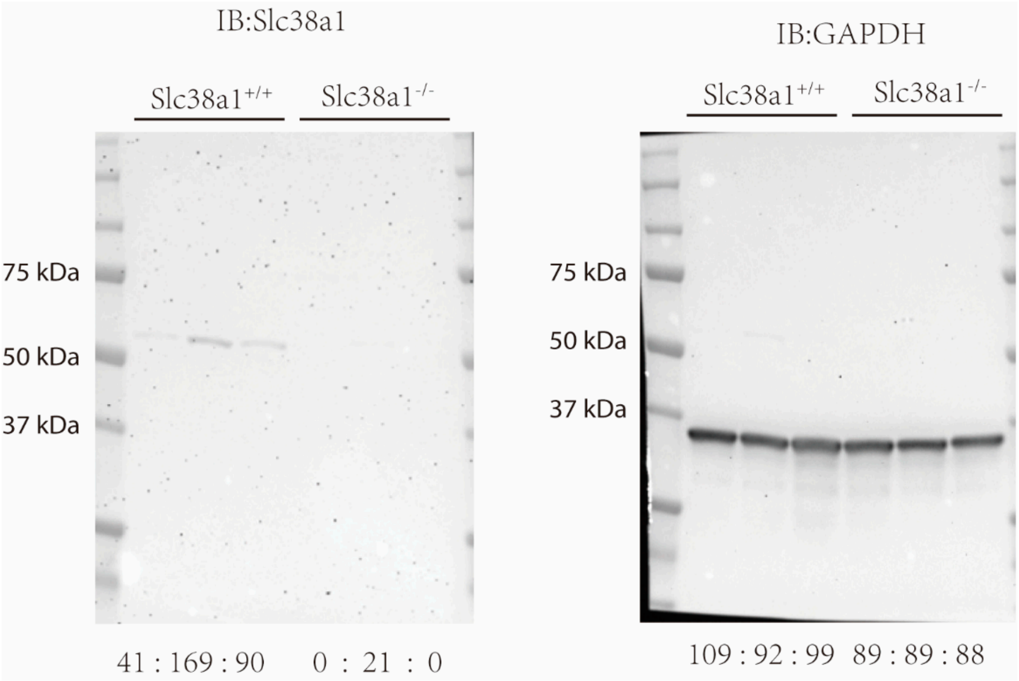

Supplement: Supplementary file 1 [file cancers-16-00405-s001.zip › cancers-2815400-supplementary.pdf]
